# BRENDA in 2019: a European ELIXIR core data resource

**DOI:** 10.1093/nar/gky1048

**Published:** 2018-11-05

**Authors:** Lisa Jeske, Sandra Placzek, Ida Schomburg, Antje Chang, Dietmar Schomburg

**Affiliations:** Braunschweig Integrated Centre of Systems Biology (BRICS), Technische Universität Braunschweig, Rebenring 56, 38106 Braunschweig, Germany

## Abstract

The BRENDA enzyme database (www.brenda-enzymes.org), recently appointed ELIXIR Core Data Resource, is the main enzyme and enzyme-ligand information system. The core database provides a comprehensive overview on enzymes. A collection of 4.3 million data for ∼84 000 enzymes manually evaluated and extracted from ∼140 000 primary literature references is combined with information obtained by text and data mining, data integration and prediction algorithms. Supplements comprise disease-related data, protein sequences, 3D structures, predicted enzyme locations and genome annotations. Major developments are a revised *ligand summary page* and the *structure search* now including a similarity and isomer search. BKMS-react, an integrated database containing known enzyme-catalyzed reactions, is supplemented with further reactions and improved access to pathway connections. In addition to existing *enzyme word maps* with graphical information of enzyme specific terms, *plant word maps* have been developed. They show a graphical overview of terms, e.g. enzyme or plant pathogen information, connected to specific plants. An *organism summary page* showing all relevant information, e.g. taxonomy and synonyms linked to enzyme data, was implemented. Based on a decision by the IUBMB enzyme task force the enzyme class EC 7 has been established for ‘translocases’, enzymes that catalyze a transport of ions or metabolites across cellular membranes.

## INTRODUCTION

BRENDA (BRaunschweig ENzyme DAtabase, www.brenda-enzymes.org), founded in 1987, is the main public information system for functional enzyme and enzyme–ligand related information. Originally, BRENDA started as a series of books (Handbook of Enzymes, (1)) and has developed to the world’s main repository on enzyme properties, used by up to 100 000 users per month. Since 1998 the website is available via the world wide web and has been evolved to an essential encyclopedia, meeting the requirements of the users in connection with the fast growing number of data (‘big data’) and new developments in the ‘OMICS’ community in the area of the systems biology, biotechnology, medical and pharmaceutical research.

The BRENDA website provides a comprehensive overview on enzymes and combines its data content with a sophisticated flexible query system with analysis and visualization tools, and data retrieval options for detailed assessment of enzyme data in more than 50 categories of enzyme properties. The contents in BRENDA encompasses data on the catalyzed reaction, nomenclature, taxonomy, enzyme–ligand interaction, inhibition, occurrence, stability, kinetics, mutants, application, protein sequence and structure, disease-related data, etc.

The data collection is based on the EC classification system of the IUBMB (International Union of Biochemistry and Molecular Biology, (2)), and the main core contains 4.3 million manually annotated experimental enzyme data of 84 000 enzymes from all taxonomic groups, evaluated and extracted by scientific experts from ∼140 000 primary literature references. Each entry is linked to its literature reference and the organism of origin. For a complete overview on the occurrence of characterized enzymes BRENDA integrates information retrieved by text mining of literature abstracts. This set of data contains 1.6 million entries from ∼3.6 million references including information on enzyme/diseases, organisms/tissues, cellular and subcellular localization and kinetic values (3,4). These data are represented in the accessory modules AMENDA, FRENDA, DRENDA and KENDA.

The BRENDA ‘ligands’ are a fundamental part of the repository, covering all compounds interacting with enzymes, such as substrates and products, cofactors, inhibitors, activating compounds, etc. stored in the associated ‘ligand’ database. Approximately 205 000 enzyme ligands (small molecules as well as macromolecules) are stored with 1.6 million functional and structural data, which can be searched and displayed.

The BRENDA Tissue Ontology (BTO) has been developed as a structured comprehensive encyclopedia of tissue terms from multicellular organisms (5). The BTO offers a direct access to information about enzymes, isolated or detected in organs, tissues, cell types and cell lines. Additionally, the human anatomy atlas CAVEman (6,7) is linked to the BTO terms providing a connection between anatomical and functional enzyme data. Along with the BTO, the subcellular localization of enzyme activity is linked to the entries of Gene Ontology (8). Furthermore, the BRENDA website provides the access to supplemental enzyme-related data, e.g. the *TaxTree Explorer*, showing all organisms of the NCBI taxonomy database (9), the *EC Explorer* representing the hierarchical EC classification of the IUBMB, both directly linked to the BRENDA *enzyme summary pages*. The 3D-structural classification of enzyme proteins is covered by SCOPe and CATH (10,11).

The EnzymeDetector was integrated into BRENDA in 2011 (12) and was substantially improved in 2014 offering an automatic comparison, evaluation and prediction of enzyme function annotations for bacterial and archaeal genomes.

In addition to the supplement data, BRENDA provides data and information of external databases, e.g. protein sequences (UniProt, (13)), 3D structures (PDB, (14)), KEGG and MetaCyc pathways (15,16), genome annotations in the Genome Explorer (17) and links to PubMed (18) for the literature references.

Since the last publication in 2017 new developments and major improvements are implemented in BRENDA. The *ligand summary page* (19) is upgraded and endowed with new functions, new links and user-optimized features. The chemical substructure search (20) is essentially revised and extended with new search options, more information fields and new links, as well as an optimized display of the search results. The BKMS-react database is substantially improved, supplemented with further reactions and a new access to pathway connections. In addition to the existing word maps, which provide graphical information of terms associated with specific enzymes, new *plant word maps* have been implemented. They show a graphical overview of terms, e.g. enzyme or plant pathogen information connected to specific plants. In this context a new *organism summary page* showing all relevant information, e.g. taxonomy and synonyms linked to the enzyme data, was implemented. The 3D protein structures in BRENDA are now linked to DoGSiteScorer and Protoss (21). These two approaches predict the druggablity and the protonation states of proteins, respectively.

## BRENDA - A EUROPEAN ELIXIR CORE DATA RESOURCE

In June 2018, BRENDA was selected by ELIXIR as a Core Data Resource, the prestigious list of databases which are critically important for life science research (22). ELIXIR is an intergovernmental organization that brings together high-quality life science resources from across Europe to ensure the long-term preservation of life science data. ELIXIR coordinates life science resources and develops ways to store, analyze and exchange data, and implement best practices. It provides platforms for computing and tools, data storage and transfer, as well as training. ELIXIR also establishes Europe-wide standards that can be used to describe life science data (https://www.elixir-europe.org/platforms/data/core-data-resources). BRENDA is also member of de.NBI, the German Network for Bioinformatics Infrastructure (https://www.denbi.de).

## NEW DEVELOPMENTS AND MAJOR IMPROVEMENTS

### The ligand summary page

In BRENDA all compounds playing a role in enzyme-catalyzed reactions (e.g. substrates, products, activators, inhibitors and cofactors) are referred to as ‘ligands’. The information about each ligand is combined and presented on the *ligand summary page*.

In addition to structural information like a graphical structure diagram, a molecular formula and the InChIKey, sets of synonyms and a representative name of the ligand are given at the top of the page. A link to all known pathways associated with the ligand is located below this basic ligand information.

**Figure 1.**
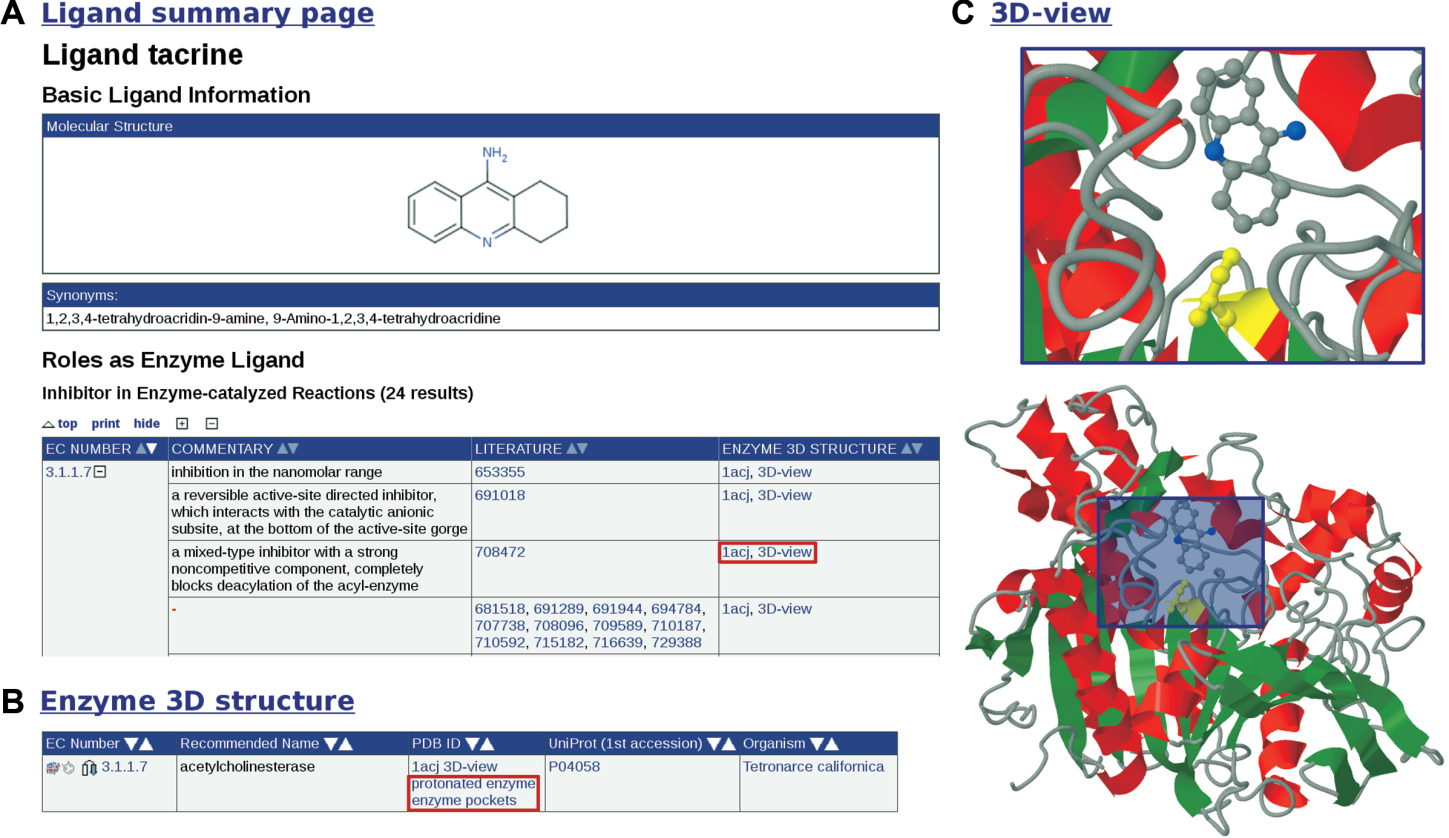
(**A**) The *ligand summary page* of acetylcholinesterase inhibitor tacrine containing the inhibitor table linked with enzyme 3D structures. The user can click at the (+)-icon to show the specific data of the EC number 3.1.1.7 or click at the (−)-icon to hide it. (**B**) 3D-structure search result page of acetylcholinesterase accessible via the entry page using the ‘Classic View’. (**C**) 3D enzyme structure of acetylcholinesterase in combination with tacrine.

The respective data fields of the ligand roles mentioned above are linked to an EC number, a reference and an enzyme 3D structure (see Figure 1A). Enzyme kinetic parameters, such as the *k*_cat_ value, *K*_M_ value, *K*_i_ value and IC_50_ value are also listed in combination with an EC number and a reference.

The last section contains all literature references of the ligand in BRENDA as well as links to ChEBI (23) and PubChem (24).

Due to the substantial increase of data the *ligand summary pages* have recently been optimized and now present a view similar to the *enzyme summary pages*. Users can adapt the appearance of the website according to their own requirements and display only data fields of interest. Similar records in the tables are summarized and can be made visible by click or mouseover. Sorting options have been implemented for the columns. The individually chosen format of the *ligand summary page* can be printed using the new function ‘print visible entries’ in the upper right corner.

### The revised and extended BRENDA structure search

The BRENDA *structure search* is an instrument for drawing a chemical structure with the JSME molecule editor (25) and searching it in ∼134 000 different molecular structures covering ∼186 000 different compound names of the BRENDA ligand database among substrates, products, inhibitors, activating compounds and cofactors.

The search form provides three different search types. In addition to the former substructure search which identified all BRENDA molecules containing the drawn structure, a similarity and an isomer search have been implemented.

The substructure and isomer searches first carry out a fingerprint scan followed by a graph-matching algorithm. A fingerprint of a molecule is a unique identifier of the respective chemical structure. This identifier is based on all paths of atoms and bonds within the molecule with a maximal length of eight atoms. The graph matching algorithm then transforms the molecular structures into undirected graphs. Thus, a molecule is represented as a network where atoms form the nodes and bonds are the edges. Atoms and bonds of the drawn structure are compared with the BRENDA ligand structures and then matched. The pre-selection of promising molecules with the fingerprint scan is executed as the first step to reduce the number of these time-consuming graph matchings.

The substructure and isomer searches are different in the matching algorithm types. In the substructure search, a subgraph-matching algorithm is used, whereas a complete matching of the graph is calculated in the isomer search excluding the spatial arrangement of the atoms.

**Figure 2.**
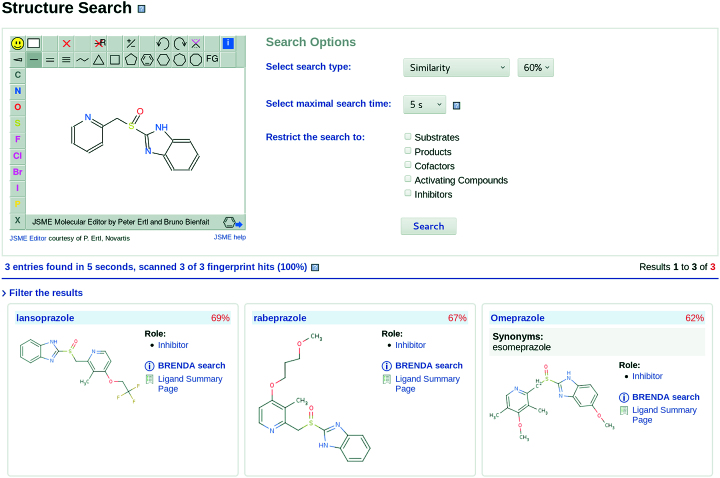
A similarity search for known proton-pump inhibitors with the parent structure.

In the similarity search only the fingerprint scan is carried out taking into account the Tanimoto coefficient (26) - a measure for similarity - and the previously user-defined minimal similarity as lower boundary. In Figure 2, a similarity search for known proton-pump inhibitors with the parent structure and a minimal similarity of 60% is displayed.

As the result, the user gets a list of all matching ligands with information about synonyms, their roles in enzyme-catalyzed reactions and the structure diagrams. If one of these ligands is of particular interest, it is possible to start a BRENDA search or visit the respective *ligand summary page* for more information, such as pathways, associated EC numbers, reaction equations, references, enzyme kinetic parameters, the molfile and the InChIKey.

In addition to the new search types the search form and result pages have been more clearly arranged. Since the matching algorithms are very time-consuming, the user can select a maximal search time. A progress bar with notifications informs about the current status of the process. Before calculations the BRENDA ligands are sorted by their number of atoms. The result table first displays the most similar structures. Additionally, the results can be filtered according to the occurrence of the respective molecules in living organisms or their involvement in enzyme classes (EC numbers).

### The integrated biochemical reaction database BKMS-react

**Figure 3.**
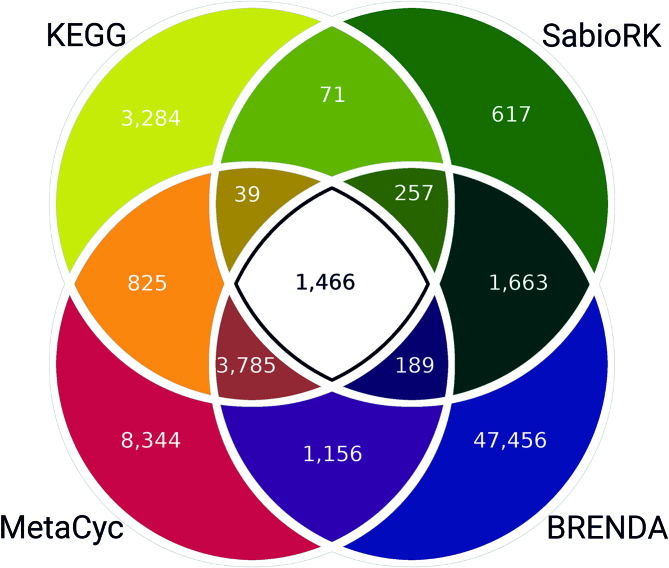
Distribution of unique reactions between BRENDA, KEGG, MetaCyc and SABIO-RK, not shown are the number of common reactions of BRENDA/KEGG and MetaCyc/SABIO-RK.

The creation of complete and reliable metabolic models requires associated data of the scientific literature for reactions. This information can be extracted from curated biological databases, such as BRENDA, KEGG, MetaCyc and SABIO-RK (27). Since the amount and annotation of reactions differ between these sources, the easy and simultaneous access to all databases via the BRENDA module BKMS-react is a great advantage. BKMS-react comprises 69 981 unique reactions and is based on a matching algorithm integrating all reactions of the mentioned databases by a comparison of compound structures and names. The distribution of unique reactions between the databases of the release 2018.2 is illustrated in Figure 3. A total of 806 reactions occur in BRENDA and KEGG and 23 reactions in MetaCyc and SABIO-RK (not shown in Figure 3). BRENDA reactions provide the largest part with a total of 56 778 unique reactions of which 47 456 can only be found in BRENDA.

**Figure 4.**
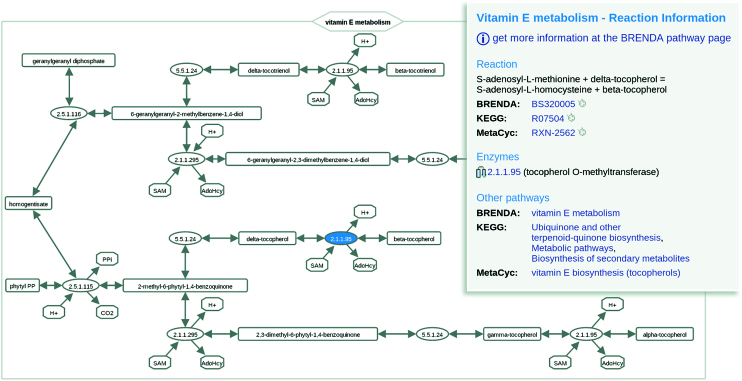
A reaction specific pathway view of the vitamin E metabolism in BKMS-react.

Recently, the original website BKM-react was supplemented with reactions of the biochemical reaction kinetics database SABIO-RK and renamed BKMS-react. Additionally, the new tab ‘Pathways’ provides a quick overview on all pathways of BRENDA, KEGG and MetaCyc associated with at least one reaction of BKMS-react. After choosing a specific pathway the respective reactions appear in the result table. Furthermore, a reaction-specific pathway view with a blue highlighted EC number node can be retrieved by clicking at a BRENDA pathway in the reaction entry (see Figure 4).

### Organism word maps and organism summary pages

**Figure 5.**
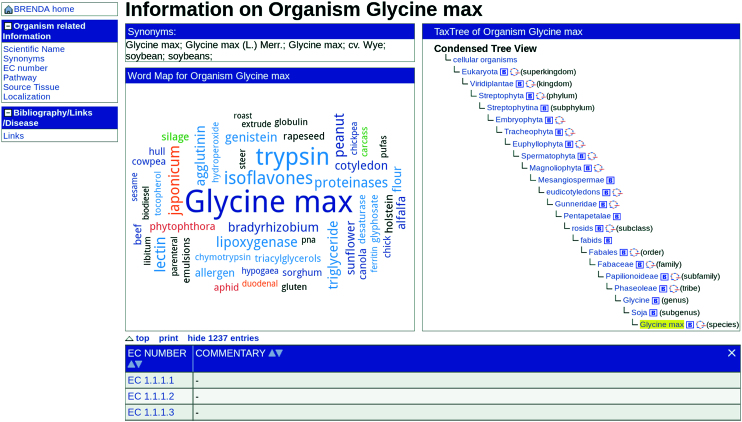
Organism summary page of *Glycine max*.

In 2015 BRENDA introduced the enzyme word maps providing a quick overview on enzyme research areas as found in the literature. Now, BRENDA provides a wider spectrum with the new *organism word maps*. The first beta version of the *organism word maps* as well as the *organism summary pages* are available for the taxonomic groups of Archaea, Bacteria, Fungi as well as Viridiplantae and will be extended to all taxonomic groups. This new option offers a quick and easy access of relevant facts published in PubMed. The terms are classified with respect to the enzyme, organism, human disease, plant disease, plant trait, plant pathogen, useful organism and habitat. In the same manner, specified for the *enzyme word maps* the information is categorized in different colors and font sizes depending on their relevance, category and specificity. Within a word map the entries are directly linked to available enzyme-related information and to the new *organism summary pages* (Figure 5).

The *organism summary page* provides the access to a wide range of information about each organism, including the scientific name, synonyms, enzymes, pathways, source tissue and subcellular localization connected to the BRENDA data. The classification within the taxonomic tree is displayed and linked directly to the NCBI Taxonomy, PubMed and to the NCBI Genome are given.

### Enzyme 3D structures linked with Protoss and DoGSiteScorer

Links to DoGSiteScorer (28) (‘enzyme pockets’) and Protoss (29) (‘protonated enzyme’), developed by the Rarey group at the Center of Bioinformatics, University of Hamburg were integrated into the 3D-structure search result pages in release 2017.1. An example is given for acetylcholinesterase as illustrated in Figure 1B. Acetylcholinesterase catalyzes the degradation of the neurotransmitter acetylcholine. A 3D image of the enzyme structure with the active site in yellow and the inhibitor tacrine in gray/blue is shown by clicking at ‘3D-view’ (see Figure 1C). Tacrine is a known acetylcholinesterase inhibitor used for symptomatic treatment of Alzheimer’s disease (30).

DoGSiteScorer is a software for druggability predictions based on the detection of potential binding pockets. For predicted pockets, the size, shape, chemical features and a druggability score are provided.

Protoss completes the hydrogen bonding network of protein–ligand complexes by adding missing hydrogen atoms. Protonation states, tautomers, hydrogen coordinates, metal interactions and repulsive forces between atoms are considered in this process.

### DRENDA - disease information on enzymes

Enzymes play an important and often essential role in the development or treatment of many diseases, either as causing agents (malfunction), used for diagnosis, or during treatment as a drug target (e.g. in infection and many others). By a combination of text mining in titles and abstracts of papers cited in PubMed and supervised classification by support vector machines, 844 260 journal articles were identified that cover enzyme and disease aspects. A total of 495 579 of them are describing causal interactions, 423 256 diagnostic applications and 330 545 roles of enzymes in therapy. By choosing a confidence level the user can either select a high precision or a high recall of the results. A total of 890 842 enzyme disease relations were identified. A combined search based on disease, enzyme and title contents allows a highly specific search for relevant papers (31).

Additionally, the MESH ontology (32) in form of a tree viewer has been added to the user interface. This provides a fast overview on the enzymes involved in a certain disease. An ontology is a presentation of terms and their logical relationships. The ontology explorer in BRENDA comprises a variety of 35 biologically and biochemically relevant ontologies. The MeSH Ontology, a hierarchical terminology for indexing biomedical literature, is such an ontology used for the classification of PubMed papers. The terms of the branch ‘Diseases’ are used in DRENDA (Disease Related ENzyme information DAtabase).

### New contents in BRENDA

The current database release (August 2018) covers 7590 enzyme classes (EC numbers), including 292 deleted, 610 transferred and 498 preliminary classes. Since September 2016, the number of EC classes increased by 598, of which 110 originated from transfers of older classes. Transfers are discussed and decided by the IUBMB enzyme task force with active participation of the BRENDA team. They become necessary when newly published enzyme data prove that the enzyme acts in a different way than originally described.

Since our last publication (33) the data for 3236 EC classes were updated. New information for the ∼60 data fields was added by manual annotation of ∼9500 additional literature references. This information characterizes each enzyme with respect to its catalyzed reactions and specificity, kinetic properties, experimental conditions, stability and sensitivity to inhibitory agents. The current data content for a selection of data fields is shown in Table 1. Special emphasis in recent data annotation was laid upon enzymes degrading polysaccharides and lignin in plant materials. Biotechnological processes of biofuel production from natural sources have gained much scientific interest. Thus, extensive data were added to the respective enzyme classes (e.g. cellulases, glycohydrolases). Suitable enzymes were found in fungi, bacteria and some archaea. Among the bacterial and archaeal enzymes, some candidates show a good stability in the harsh conditions, which may occur during biofuel processing. The fungal enzymes however show a broader substrate spectrum, including branched polysaccharides which are part of the hemicelluloses. Unfortunately, these enzymes are rarely active above 60°C. A literature search for fungal habitats with elevated temperatures retrieved only a few species from the genus *Thermomyces*.

**Table 1.** Number of entries in selected data fields

Enzyme information	Entries 2016	Entries 2018
Substrates and products	407 446	435 289
Inhibitors	196 548	207 441
Cofactors	14 382	15 964
Metals and ions	36 711	38 548
Activating compounds	26 761	27 653
*K*_M_-Values	135 603	145 215
*K*_i-_Values	38 378	39 927
*k*_cat_-Values	62 445	68 963
Specific activity	45 773	48 001
IC_50_- Values	49 842	54 230
Localization and source/tissue	96 889	102 758
Enzyme names and synonyms	102 394	111 488
Citations (manually annotated)	146 221	155 422
Isolation and preparation/crystallization	88 849	96 765
Enzyme structure	158 397	196 071
Mutant enzymes	76 451	83 355
Enzyme stability	47 281	49 271
Enzyme application	15 080	16 441

The numbers refer to the combination of enzyme protein, source organism and literature reference. The term enzyme protein refers either to a protein sequence or to a protein isolated from a given organism without its sequence having been determined.

### New enzyme class 7 - an important innovation of the nomenclature system

In 1961, the enzyme commission of the International Union of Biochemistry established the fundamental scheme of enzyme classification based on the catalyzed reactions. Six main classes were established at that time. Sub- and sub-subclasses have been added since, but no changes had been necessary regarding the main classes. With the new EC class 7 this will now be a major change in the EC system of enzyme classification.

For cell growth and metabolism, all organisms need enzymes for transporting compounds across cellular membranes. Several of these transports are driven by the hydrolysis of adenosine triphosphate and the enzymes were accordingly classified as hydrolases in EC 3.6.3.-. However, their primary function is the transport across a membrane and not the hydrolytic reaction. A new EC main class 7 named ‘Translocases’ was recently established by the IUBMB. The reactions catalyzed are designated as transfers from ‘side 1’ to ‘side 2’. Older designations like ‘in’ and ‘out’ (or ‘cis’ and ‘trans’) were discarded since they can be ambiguous. The new class and its currently populated subclasses are defined as follows:

EC 7 Translocases
  EC 7.1 Catalyzing the translocation of hydrons
    EC 7.1.1 Hydron translocation or charge separation linked to oxidoreductase reactions
    EC 7.1.2 Hydron translocation linked to the hydrolysis of a nucleoside triphosphate
    EC 7.1.3 Hydron translocation linked to the hydrolysis of diphosphate
  EC 7.2 Catalyzing the translocation of inorganic cations
    EC 7.2.1 Linked to oxidoreductase reactions
    EC 7.2.2 Linked to the hydrolysis of a nucleoside triphosphate
    EC 7.2.3 Hydron translocation linked to the hydrolysis of diphosphate
    EC 7.2.4 Linked to decarboxylation
  EC 7.3 Catalyzing the translocation of inorganic anions and their chelates
    EC 7.3.2 Linked to the hydrolysis of a nucleoside triphosphate
  EC 7.4 Catalyzing the translocation of amino acids and peptides
    EC 7.4.2 Linked to the hydrolysis of a nucleoside triphosphate
  EC 7.5 Catalyzing the translocation of carbohydrates and their derivatives
    EC 7.5.2 Linked to the hydrolysis of a nucleoside triphosphate
  EC 7.6 Catalyzing the translocation of other compounds
    EC 7.6.2 Linked to the hydrolysis of a nucleoside triphosphate

The transfer of the translocases from EC 3.6.3.- is performed in batches of 10 to 30 entries, which are published by the IUBMB (http://www.enzyme-database.org/newenz.php). The corresponding BRENDA data are made public twice yearly in the regular BRENDA releases. The first set of transferred translocases will appear in BRENDA in February 2019.

## DATA AVAILABILITY

All described databases and features are accessible via the main BRENDA website: https://www.brenda-enzymes.org/. The biochemical reaction database BKMS-react can also be accessed via http://bkms-react.tu-bs.de/.
